# Poly[bis­[μ_2_-(dimethyl­aza­nium­yl)methyl­enediphospho­nato]magnesium]

**DOI:** 10.1107/S1600536811005976

**Published:** 2011-02-23

**Authors:** Qiao-Sheng Hu, Xiao-Yu Deng, Yu-Hui Sun, Zi-Yi Du

**Affiliations:** a College of Chemistry and Life Science, Gannan Normal University, Ganzhou, Jiangxi 341000, People’s Republic of China

## Abstract

The title compound, [Mg(C_3_H_10_NO_6_P_2_)_2_]_*n*_, synthesized by a hydro­thermal method, adopts a one-dimensional polymeric chain structure and is isotypic with the previously reported Cd complex based on the ligand *N*,*N*-dimethyl­amino­methane-1,1-diphospho­nic acid (H_4_ *L*). The asymmetric unit contains one half Mg^2+^ ion and one H_3_ *L* ^−^ anion. The unique Mg^2+^ ion lies on an inversion center and is octa­hedrally coordinated by O atoms from six phospho­nate groups of four different H_3_ *L* ^−^ anions. Each H_3_ *L* ^−^ anion, with one protonated N atom and two phospho­nate OH groups, serves as a tridentate ligand. Two of its six phospho­nate O atoms chelate to a Mg^2+^ cation in a bidentate fashion, while a third O atom bridges to a neighbouring Mg^2+^ ion. The inter­connection of Mg^2+^ ions by the H_3_ *L* ^−^anions leads to the formation of a polymer chain along the *a* axis in which the adjacent Mg^2+^ ions are doubly bridged by two equivalent H_3_ *L* ^−^ anions. These discrete chains are further assembled into a three-dimensional supra­molecular network *via* O—H⋯O and N—H⋯O hydrogen bonds involving the non-coordin­ated phospho­nate O atoms and the protonated N atoms.

## Related literature

For other metal complexes based on the *N*,*N*-dimethyl­amino­methane-1,1- diphospho­nate ligand, see: Du *et al.* (2009 ▶, 2010*a* ▶,*b* ▶). For bond-length data, see: Lutz & Muller (1995 ▶); Distler *et al.* (1999 ▶); Stock & Bein (2004 ▶). 
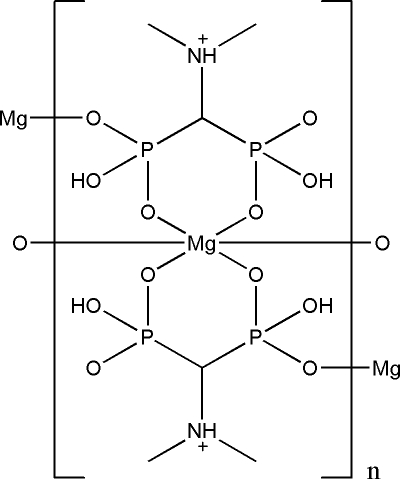


## Experimental

### 

#### Crystal data

[Mg(C_3_H_10_NO_6_P_2_)_2_]
*M* *_r_* = 460.43
Monoclinic, 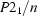

*a* = 5.4507 (3) Å
*b* = 11.2166 (6) Å
*c* = 12.5770 (7) Å
β = 94.984 (1)°
*V* = 766.03 (7) Å^3^
*Z* = 2
Mo *K*α radiation
μ = 0.61 mm^−1^
*T* = 296 K
0.40 × 0.30 × 0.24 mm

#### Data collection

Bruker SMART APEX CCD area-detector diffractometer
Absorption correction: multi-scan (*SADABS*; Bruker, 2008 ▶) *T*_min_ = 0.675, *T*_max_ = 0.746
4801 measured reflections
1492 independent reflections
1447 reflections with *I* > 2σ(*I*)
*R*_int_ = 0.014

#### Refinement

*R*[*F* ^2^ > 2σ(*F* ^2^)] = 0.024
*wR*(*F* ^2^) = 0.066
*S* = 1.09
1492 reflections
115 parameters
H-atom parameters constrained
Δρ_max_ = 0.38 e Å^−3^
Δρ_min_ = −0.33 e Å^−3^

### 

Data collection: *SMART* (Bruker, 2008 ▶); cell refinement: *SAINT* (Bruker, 2008 ▶); data reduction: *SAINT*; program(s) used to solve structure: *SHELXS97* (Sheldrick, 2008 ▶); program(s) used to refine structure: *SHELXL97* (Sheldrick, 2008 ▶); molecular graphics: *SHELXTL* (Sheldrick, 2008 ▶) and *DIAMOND* (Brandenburg, 1999 ▶); software used to prepare material for publication: *SHELXTL*.

## Supplementary Material

Crystal structure: contains datablocks I, global. DOI: 10.1107/S1600536811005976/sj5102sup1.cif

Structure factors: contains datablocks I. DOI: 10.1107/S1600536811005976/sj5102Isup2.hkl

Additional supplementary materials:  crystallographic information; 3D view; checkCIF report

## Figures and Tables

**Table 1:** Hydrogen-bond geometry (Å, °)

*D*—H⋯*A*	*D*—H	H⋯*A*	*D*⋯*A*	*D*—H⋯*A*
N1—H1*B*⋯O3	0.91	2.57	3.0997 (18)	118
N1—H1*B*⋯O4^i^	0.91	2.31	3.1346 (18)	151
O3—H3*D*⋯O6^ii^	0.82	1.70	2.5011 (16)	166
O4—H4*A*⋯O2^iii^	0.82	1.81	2.6037 (16)	163

Symmetry codes: (i) 
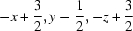
; (ii) 
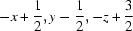
; (iii) 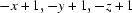.

**Fig. 1.**
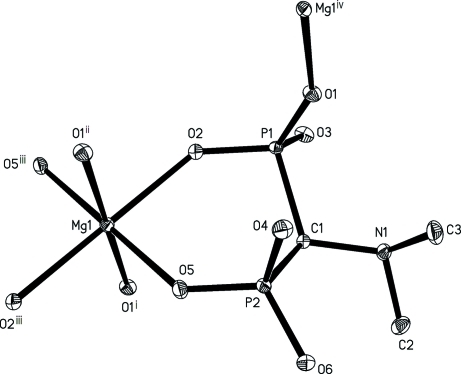
A view of the selected unit of (I), showing the atom-numbering scheme. Displacement ellipsoids are drawn at the 30% probability level. Hydrogen atoms have been omitted for clarity. [Symmetry codes: (i) x - 1, y, z; (ii) -x + 1, -y + 1, -z + 1; (iii) -x, -y + 1, -z + 1; (iv) x + 1, y, z.]

**Fig. 2.**
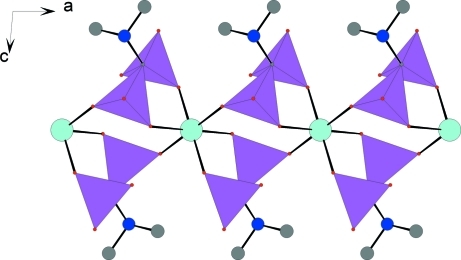
A view of the chain structure of (I) along the a-axis. The CPO3 tetrahedra are shaded in purple. Mg, N and C atoms are drawn as cyan, blue and grey circles, respectively. Hydrogen atoms have been omitted for clarity.

**Fig. 3.**
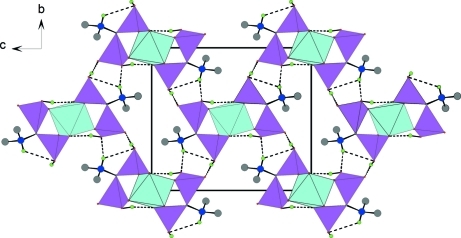
A view of the three-dimensional supramolecular structure of (I) down the a-axis. The MgO6 octahedra and CPO3 tetrahedra are shaded in cyan and purple, respectively. N, C and H atoms are drawn as blue, grey and green circles, respectively. Hydrogen bonds are represented by dashed lines.

## supplementary crystallographic information

### Comment

Among many of the phosphonate ligands studied so far, methylenediphosphonic acid and its derivatives are quite unique because they feature a close connection of two phosphonate moieties *via* one carbon atom, which facilitate their combined coordination ability to act as a [CP_2_O_6_] unit rather than two [CPO_3_] units. As a result, they show diversified coordination capabilities with metal ions and thus lead to the formation of new structural types. Recently, by using such ligand types, *i.e. N*,*N*-dimethylaminomethane-1,1-diphosphonate, we have isolated a series of diphosphonate complexes of metals such as Al^III^, Fe^III^, Cd^II^, Pb^II^ and Ba^II^, which exhibit variable structures such as zero-dimensional, one-dimensional, double-1-dimensional, double-2-dimensional, and three-dimensional structures (Du *et al.*, 2009, 2010*a*,*b*). As an expansion of our previous work, we have also obtained a one-dimensional magnesium(II) diphosphonate, namely [Mg(C_6_H_20_N_2_O_12_P_4_)]_n_, which is isostructural with the previously reported cadmium(II) complex based on the same ligand and shows a one-dimensional chain structure. The asymmetric unit contains a half Mg^2+^ cation and one H_3_*L*^-^ anion. The unique Mg^2+^ cation lies on an inversion center and is octahedrally coordinated by the O atoms of six phosphonate groups from four H_3_*L*^-^ anions. The Mg—O [2.0448 (11) – 2.1879 (11) Å] bond lengths are comparable to those reported for other Mg^II^ phosphonate complexes (Lutz & Muller, 1995; Distler *et al.*, 1999; Stock & Bein, 2004). The unique H_3_*L*^-^ anion, with one protonated N atom and two phosphonate OH groups, serves as a tridentate ligand. By using three of its six phosphonate O atoms, it chelates in a bidentate fashion with one Mg^2+^ cation and also bridges to a second Mg^2+^ ion. The interconnection of Mg^2+^ cations by the H_3_*L*^-^ anions leads to the formation of a one-dimensional chain along the *a*-axis, in which the adjacent Mg^2+^ ions are doubly bridged by two equivalent H_3_*L*^-^ anions. These discrete one-dimensional chains are further assembled into a three-dimensional supramolecular network *via* O—H···O and N—H···O hydrogen bonds involving the non-coordinated phosphonate O atoms and the protonated N atoms.

### Experimental

For the preparation of (I), a mixture of Mg(NO_3_)_2_ (0.20 mmol) and H_4_L (0.50 mmol) and ethanol (3 ml) in 10 ml distilled water, was sealed into a Parr Teflon-lined autoclave (23 ml) and heated at 393 K for 3 d. Colorless block-shaped crystals were collected in  *ca* 55% yield based on Mg. Analysis calculated for C_6_H_20_N_2_O_12_Mg_1_P_4_: C 15.65, H 4.38, N 6.08%; found: C 15.59, H 4.48, N 6.03%.

### Refinement

The N-bound and the tertiary C-bound H atoms were positioned geometrically and refined using a riding model: N—H = 0.91 and C—H = 0.98 Å, with *U*iso(H) = 1.2*U*_eq_(N, C); while the O-bound and the primary C-bound H atoms were placed in idealized positions and constrained to ride on their parent atoms: O—H = 0.82 and C—H = 0.96 Å, with *U*_iso_(H) = 1.5 times *U*_eq_(O, C).

### Crystal data

**Table:**

[Mg(C_3_H_10_NO_6_P_2_)_2_]	*F*(000) = 476
*M**_r_* = 460.43	*D*_x_ = 1.996 Mg m^−^^3^
Monoclinic, *P*2_1_/*n*	Mo *K*α radiation, λ = 0.71073 Å
Hall symbol: -P 2yn	Cell parameters from 4647 reflections
*a* = 5.4507 (3) Å	θ = 2.4–29.4°
*b* = 11.2166 (6) Å	µ = 0.61 mm^−^^1^
*c* = 12.5770 (7) Å	*T* = 296 K
β = 94.984 (1)°	Block, colourless
*V* = 766.03 (7) Å^3^	0.40 × 0.30 × 0.24 mm
*Z* = 2	

### Data collection

**Table:**

Bruker SMART APEX CCD area-detector diffractometer	1492 independent reflections
Radiation source: fine-focus sealed tube	1447 reflections with *I* > 2σ(*I*)
graphite	*R*_int_ = 0.014
φ and ω scans	θ_max_ = 26.0°, θ_min_ = 2.4°
Absorption correction: multi-scan (*SADABS*; Bruker, 2008)	*h* = −6→6
*T*_min_ = 0.675, *T*_max_ = 0.746	*k* = −13→13
4801 measured reflections	*l* = −15→15

### Refinement

**Table:**

Refinement on *F*^2^	Primary atom site location: structure-invariant direct methods
Least-squares matrix: full	Secondary atom site location: difference Fourier map
*R*[*F*^2^ > 2σ(*F*^2^)] = 0.024	Hydrogen site location: inferred from neighbouring sites
*wR*(*F*^2^) = 0.066	H-atom parameters constrained
*S* = 1.09	*w* = 1/[σ^2^(*F*_o_^2^) + (0.0347*P*)^2^ + 0.6187*P*] where *P* = (*F*_o_^2^ + 2*F*_c_^2^)/3
1492 reflections	(Δ/σ)_max_ = 0.001
115 parameters	Δρ_max_ = 0.38 e Å^−^^3^
0 restraints	Δρ_min_ = −0.33 e Å^−^^3^

### Special details

**Table:**

Experimental. IR data (KBr, ν, cm^-1^): 3437 (*m*), 3137 (*s*), 3071 (*m*), 2986 (*m*), 2826 (*m*), 2280 (*m*), 1815 (*m*), 1473 (*m*), 1457 (*m*), 1421 (*m*), 1388 (*m*), 1256 (*s*), 1225 (*s*), 1200 (*versus*), 1155 (*s*), 1128 (*s*), 1088 (*s*), 1036 (*s*), 995 (*s*), 950 (*s*), 928 (*s*), 854 (*m*), 827 (*m*), 725 (*m*), 615 (*m*), 573 (*s*), 517 (*m*), 476 (*m*).
Geometry. All e.s.d.'s (except the e.s.d. in the dihedral angle between two l.s. planes) are estimated using the full covariance matrix. The cell e.s.d.'s are taken into account individually in the estimation of e.s.d.'s in distances, angles and torsion angles; correlations between e.s.d.'s in cell parameters are only used when they are defined by crystal symmetry. An approximate (isotropic) treatment of cell e.s.d.'s is used for estimating e.s.d.'s involving l.s. planes.
Refinement. Refinement of *F*^2^ against ALL reflections. The weighted *R*-factor *wR* and goodness of fit *S* are based on *F*^2^, conventional *R*-factors *R* are based on *F*, with *F* set to zero for negative *F*^2^. The threshold expression of *F*^2^ > σ(*F*^2^) is used only for calculating *R*-factors(gt) *etc*. and is not relevant to the choice of reflections for refinement. *R*-factors based on *F*^2^ are statistically about twice as large as those based on *F*, and *R*- factors based on ALL data will be even larger.

### Fractional atomic coordinates and isotropic or equivalent isotropic displacement parameters (Å^2^)

**Table:**

	*x*	*y*	*z*	*U*_iso_*/*U*_eq_	
Mg1	0.0000	0.5000	0.5000	0.01237 (17)	
P1	0.52519 (7)	0.34374 (3)	0.59421 (3)	0.01109 (12)	
P2	0.34788 (7)	0.55738 (3)	0.72234 (3)	0.01225 (13)	
N1	0.5807 (3)	0.35032 (12)	0.81729 (11)	0.0161 (3)	
H1B	0.6307	0.2757	0.8007	0.019*	
C1	0.4187 (3)	0.39596 (14)	0.72149 (12)	0.0126 (3)	
H1A	0.2596	0.3565	0.7262	0.015*	
C2	0.4447 (4)	0.33952 (16)	0.91574 (13)	0.0217 (4)	
H2A	0.5545	0.3102	0.9737	0.032*	
H2B	0.3096	0.2851	0.9024	0.032*	
H2C	0.3831	0.4163	0.9341	0.032*	
C3	0.8076 (3)	0.42374 (19)	0.84083 (15)	0.0264 (4)	
H3A	0.9027	0.3918	0.9021	0.040*	
H3B	0.7620	0.5045	0.8550	0.040*	
H3C	0.9041	0.4221	0.7805	0.040*	
O1	0.7774 (2)	0.38860 (10)	0.57970 (9)	0.0168 (3)	
O2	0.3180 (2)	0.38023 (10)	0.51279 (9)	0.0160 (2)	
O3	0.5417 (2)	0.20563 (10)	0.60652 (10)	0.0182 (3)	
H3D	0.4114	0.1800	0.6253	0.027*	
O4	0.5780 (2)	0.62560 (11)	0.68532 (9)	0.0177 (3)	
H4A	0.5846	0.6161	0.6210	0.027*	
O5	0.1277 (2)	0.57125 (10)	0.64393 (9)	0.0167 (3)	
O6	0.3209 (2)	0.59441 (11)	0.83501 (9)	0.0187 (3)	

### Atomic displacement parameters (Å^2^)

**Table:**

	*U*^11^	*U*^22^	*U*^33^	*U*^12^	*U*^13^	*U*^23^
Mg1	0.0099 (4)	0.0144 (4)	0.0127 (4)	−0.0013 (3)	0.0006 (3)	0.0007 (3)
P1	0.0111 (2)	0.0108 (2)	0.0117 (2)	−0.00042 (14)	0.00234 (15)	0.00033 (14)
P2	0.0116 (2)	0.0127 (2)	0.0124 (2)	0.00185 (14)	0.00060 (15)	−0.00118 (14)
N1	0.0179 (7)	0.0157 (7)	0.0144 (6)	0.0044 (5)	−0.0008 (5)	0.0006 (5)
C1	0.0116 (7)	0.0146 (7)	0.0114 (7)	0.0011 (6)	0.0004 (6)	0.0000 (6)
C2	0.0286 (10)	0.0216 (9)	0.0150 (8)	0.0014 (7)	0.0029 (7)	0.0032 (6)
C3	0.0153 (9)	0.0371 (10)	0.0257 (9)	−0.0014 (8)	−0.0047 (7)	0.0022 (8)
O1	0.0137 (6)	0.0172 (6)	0.0200 (6)	−0.0027 (4)	0.0041 (4)	0.0022 (5)
O2	0.0142 (6)	0.0197 (6)	0.0141 (5)	0.0016 (4)	0.0008 (4)	0.0009 (4)
O3	0.0178 (6)	0.0122 (6)	0.0253 (6)	−0.0009 (4)	0.0067 (5)	0.0010 (5)
O4	0.0171 (6)	0.0191 (6)	0.0171 (5)	−0.0036 (5)	0.0022 (5)	−0.0025 (5)
O5	0.0147 (6)	0.0173 (6)	0.0174 (6)	0.0022 (4)	−0.0018 (5)	−0.0004 (4)
O6	0.0196 (6)	0.0217 (6)	0.0148 (6)	0.0060 (5)	0.0015 (5)	−0.0033 (5)

### Geometric parameters (Å, °)

**Table:**

Mg1—O5^i^	2.0448 (11)	N1—C3	1.494 (2)
Mg1—O5	2.0448 (11)	N1—C2	1.502 (2)
Mg1—O1^ii^	2.0615 (11)	N1—C1	1.5196 (19)
Mg1—O1^iii^	2.0616 (11)	N1—H1B	0.9100
Mg1—O2	2.1879 (11)	C1—H1A	0.9800
Mg1—O2^i^	2.1879 (11)	C2—H2A	0.9600
P1—O1	1.4898 (12)	C2—H2B	0.9600
P1—O2	1.5134 (12)	C2—H2C	0.9600
P1—O3	1.5587 (12)	C3—H3A	0.9600
P1—C1	1.8451 (15)	C3—H3B	0.9600
P2—O5	1.4938 (12)	C3—H3C	0.9600
P2—O6	1.4961 (12)	O1—Mg1^iv^	2.0615 (11)
P2—O4	1.5737 (12)	O3—H3D	0.8200
P2—C1	1.8515 (16)	O4—H4A	0.8200
			
O5^i^—Mg1—O5	179.999 (1)	C3—N1—C1	112.68 (13)
O5^i^—Mg1—O1^ii^	88.62 (5)	C2—N1—C1	112.71 (13)
O5—Mg1—O1^ii^	91.38 (5)	C3—N1—H1B	107.1
O5^i^—Mg1—O1^iii^	91.38 (5)	C2—N1—H1B	107.1
O5—Mg1—O1^iii^	88.62 (5)	C1—N1—H1B	107.1
O1^ii^—Mg1—O1^iii^	180.0	N1—C1—P1	112.08 (10)
O5^i^—Mg1—O2	91.82 (4)	N1—C1—P2	115.59 (10)
O5—Mg1—O2	88.18 (4)	P1—C1—P2	113.39 (8)
O1^ii^—Mg1—O2	84.94 (4)	N1—C1—H1A	104.8
O1^iii^—Mg1—O2	95.06 (4)	P1—C1—H1A	104.8
O5^i^—Mg1—O2^i^	88.19 (4)	P2—C1—H1A	104.8
O5—Mg1—O2^i^	91.82 (4)	N1—C2—H2A	109.5
O1^ii^—Mg1—O2^i^	95.06 (4)	N1—C2—H2B	109.5
O1^iii^—Mg1—O2^i^	84.94 (4)	H2A—C2—H2B	109.5
O2—Mg1—O2^i^	180.00 (6)	N1—C2—H2C	109.5
O1—P1—O2	117.90 (7)	H2A—C2—H2C	109.5
O1—P1—O3	107.58 (7)	H2B—C2—H2C	109.5
O2—P1—O3	111.67 (7)	N1—C3—H3A	109.5
O1—P1—C1	111.24 (7)	N1—C3—H3B	109.5
O2—P1—C1	103.22 (7)	H3A—C3—H3B	109.5
O3—P1—C1	104.42 (7)	N1—C3—H3C	109.5
O5—P2—O6	117.19 (7)	H3A—C3—H3C	109.5
O5—P2—O4	111.67 (7)	H3B—C3—H3C	109.5
O6—P2—O4	106.96 (7)	P1—O1—Mg1^iv^	148.50 (8)
O5—P2—C1	104.71 (7)	P1—O2—Mg1	139.08 (7)
O6—P2—C1	108.34 (7)	P1—O3—H3D	109.5
O4—P2—C1	107.57 (7)	P2—O4—H4A	109.5
C3—N1—C2	109.91 (14)	P2—O5—Mg1	137.38 (7)

Symmetry codes: (i) −*x*, −*y*+1, −*z*+1; (ii) −*x*+1, −*y*+1, −*z*+1; (iii) *x*−1, *y*, *z*; (iv) *x*+1, *y*, *z*.

### Hydrogen-bond geometry (Å, °)

**Table:**

*D*—H···*A*	*D*—H	H···*A*	*D*···*A*	*D*—H···*A*
N1—H1B···O3	0.91	2.57	3.0997 (18)	118
N1—H1B···O4^v^	0.91	2.31	3.1346 (18)	151
O3—H3D···O6^vi^	0.82	1.70	2.5011 (16)	166
O4—H4A···O2^ii^	0.82	1.81	2.6037 (16)	163

Symmetry codes: (v) −*x*+3/2, *y*−1/2, −*z*+3/2; (vi) −*x*+1/2, *y*−1/2, −*z*+3/2; (ii) −*x*+1, −*y*+1, −*z*+1.
